# Draft Genome Sequences of Two Closely Related *Marichromatium* Isolates, Photosynthetic Gammaproteobacteria from Marine Ecosystems

**DOI:** 10.1128/MRA.01510-18

**Published:** 2019-01-10

**Authors:** Michael S. Guzman, Jonathan Chris Kuse, Natalia Santiago-Merced, Arpita Bose

**Affiliations:** a Department of Biology, Washington University in St. Louis, St. Louis, Missouri, USA; University of Arizona

## Abstract

*Marichromatium* species are photosynthetic gammaproteobacteria found in marine environments. Only two *Marichromatium* genomes are publicly available. Here, we report the draft genome sequences of two new *Marichromatium* strains isolated from Woods Hole, MA. These strains share >99% similarity to Marichromatium gracile and M. indicum based on 16S rRNA gene sequence analysis.

## Abstract (precis)

*Marichromatium* species are photosynthetic gammaproteobacteria found in marine environments. Only two *Marichromatium* genomes are publicly available.

## ANNOUNCEMENT

*Marichromatium* is a genus of photosynthetic purple sulfur bacteria that are found in marine ecosystems (1). Through their metabolic activities they participate in the cycling of carbon, sulfur, nitrogen, and other elements (2–6). Currently, of the five characterized *Marichromatium* species, only two are genome sequenced and deposited in the NCBI GenBank database, M. gracile YL-28 (7) and M. purpuratum 984. To increase the number of genomes available for comparative genomics, we isolated and sequenced two *Marichromatium* isolates from the Trunk River estuary in Woods Hole, MA. Their identity was confirmed by 16S rRNA gene sequence analysis. Here, we present the draft genome sequences of these isolates, namely, *Marichromatium* sp. strains AB31 and AB32.

Seawater was sampled from the top of the water column. Seawater samples of 500 μl were transferred to Pfennig bottles containing anoxic artificial seawater medium (8) supplemented with 20 mM acetate with far-red light (∼850 nm) at 30°C. Subculturing was performed anoxically for at least six transfers followed by streaking oxically on Bacto agar with Difco marine broth 2216 (BD Diagnostic Systems, Sparks, MD). Genomic DNA was isolated with the DNeasy blood and tissue kit (Qiagen, Dusseldorf, Germany) according to the manufacturer’s recommendations from midlog-phase cultures grown in marine broth. The 16S rRNA gene sequences were amplified from genomic DNA using the universal primers 27F and 1492R (9). PCR amplicons were Sanger sequenced, and reads were assembled with MUSCLE version 3.8.31 using the default parameters (10). NCBI BLAST analysis was then performed on the 16S rRNA gene sequences to determine the identity of each strain with the BLASTN algorithm using the default parameters (11). Phylogenetic analyses were performed with these BLASTN results using the BLAST Tree View widget with the default parameters (http://blast.ncbi.nlm.nih.gov/).

Paired-end 250-bp libraries were prepared using the Nextera sample prep kit (Illumina Inc., San Diego, CA) and were sequenced on a MiSeq platform using v2 chemistry (Illumina Inc., San Diego, CA) to 41× and 33× coverage for strains AB31 and AB32, respectively. Reads were quality and adapter trimmed with Trimmomatic version 0.38 with the program’s default parameters for paired-end reads (12). Processed reads were *de novo* assembled with SPAdes version 3.13.0 using the program’s default parameters (13). Genome assembly yielded 110 and 217 contigs for strains AB31 and AB32, respectively. The total assembly was 3,777,27 bp with an *N*_50_ value of 145,600 bp for AB31 and 3,744,942 bp with an *N*_50_ value of 57,651 bp for AB32. Both genomes had a GC content of 68.4%. Functional analysis was performed with the Rapid Annotations using Subsystems Technology (RAST) server (14, 15) version 2.0 (http://rast.nmpdr.org/rast.cgi) via the RASTtk pipeline using the default parameters (16). Sequences were submitted for annotation to the NCBI Prokaryotic Genome Annotation Pipeline (PGAP) using the default parameters (17).

Strains AB31 and AB32 are 99.78% identical to Marichromatium gracile DSM 203 and Marichromatium indicum strain JA290 based on 16S rRNA gene sequence analysis. A total of 3,466 protein coding genes were identified via RAST for both AB31 and AB32. Typical genes involved in photoautotrophic and photoheterotrophic metabolism were present in each genome. In addition, numerous terminal reductases for anaerobic metabolism were identified, including a dissimilatory sulfite and a dimethyl sulfoxide reductase. These results demonstrate the metabolic potential of *Marichromatium* spp. These draft genomes also expand genomic resources within the *Marichromatium* genus.

### Data availability.

The whole-genome shotgun (WGS) projects were deposited in GenBank under the accession numbers RHFI00000000 for *Marichromatium* sp. strain AB31 and RHFJ00000000 for *Marichromatium* sp. strain AB32. Raw sequencing reads have been deposited in the NCBI Sequence Read Archive (SRA) under the accession numbers SAMN10285739 for AB31 and SAMN10285743 for AB32.
